# Subsidy policy selection of elderly care service projects under uncertain actual demand: a real options analysis based on China’s experience

**DOI:** 10.1186/s12877-021-02690-0

**Published:** 2022-01-12

**Authors:** Huan Song, Kehan Ji, Tao Sun

**Affiliations:** 1 Zhou Enlai School of Government, Nankai University, No.38 Tongyan Road, Jinnan District, Tianjin, 300350 China; 2 School of Humanities and Social Sciences, Harbin Institute of Technology (Shenzhen), Shenzhen University Town, Nanshan District, Shenzhen, 518055 China; 3 Computational Social Science Laboratory, Nankai University, No.38 Tongyan Road, Jinnan District, Tianjin, 300350 China

**Keywords:** Elderly care, Project investment, Subsidy policy, Actual demand, Uncertainty, Real options theory

## Abstract

**Background:**

Elderly care service projects (ECSPs) aim to provide care services with the help of market forces on the supply side to satisfy the huge demand of the elderly. Subsidies play an important role in motivating the investors to invest in the ECSPs immediately. The optimal subsidy scheme should balance the policy costs and the investors’ interests.

**Methods:**

Based on the policy background of China, this study applied the real options theory to compare the effects of construction subsidy and operating subsidy on achieving policy goals from the perspective of uncertain actual demand. It introduced numerical examples to identify the optimal subsidy scheme and embedded the data from the Chinese Longitudinal Healthy Longevity Survey (CLHLS) to verify the uncertainty of actual demand.

**Results:**

The results showed that in the context of uncertain actual demand, operating subsidy has greater advantages in reducing investment thresholds, saving subsidy costs and increasing spillover values. Moreover, a sound quality supervision system, a differentiated operating subsidy scheme and a sustainable growth market demand environment are conducive to increasing the long-term interests of the government and the investors.

**Conclusions:**

The study emphasized the importance of subsidy selection in the context of uncertain actual demand, and provided a practical reference for policy designers in China and other developing countries to choose the optimal subsidy scheme for the ECSPs.

## Background

With the rapidly increasing of aging population, there is a huge demand for elderly care services. It is widely recognized that the role of family as the main elderly caregiver is being weakened due to socioeconomic changes such as the miniaturization of family structure [1], the mobility of adult children [2], the change of family concept [3] and the participation of women in labour [4]. As another traditional provider of elderly care services, the state is becoming increasingly overwhelmed by the vast demand volumes. For example, the proportion of Gross Domestic Product spent on elderly care is predicted to more than double between 2000 and 2050 in the United Kingdom [5]. As a result, some developed countries such as the United States and Germany have introduced market forces by developing elderly care service projects (ECSPs) on the supply side of health and welfare system to provide diversified and professional care for the elderly, including a series of non-medical and medical services [6, 7].

Regarding the issue of elderly care caused by aging, developing countries are often described as “getting old before getting rich”. As of 2017, more than half of the world’s population aged 80 and over─the group most in need of care─lived in developing countries, and one in four of whom lived in China [8, 9]. Although China has become the world’s second largest economy, it is still a major challenge for the government to directly provide care services to satisfy the huge demand of the elderly [10]. Many developing countries currently remain in the initial stages of the construction and development of the ECSPs, including China. The Chinese government has formulated subsidy policies to encourage private investors to actively invest in the ECSPs, so as to achieve the goal of establishing a market-oriented supply system [11]. These subsidy policies mainly revolve around construction subsidy and operating subsidy, and subsidy funds are borne by local governments [11, 12].

However, the uncertainty of actual market demand increases the risk of investment, which makes many investors keep a wait-and-see attitude towards the ECSPs and take the decision to delay investment. At present, the demand of the elderly for care services is mainly manifested as potential demand, which is the maximum space and capacity of market demand. When it is transformed into actual demand in the context of marketization, it will be disturbed by many economic, social, cultural and other factors and become uncertain. For example, the price of private supply is an important factor that hinders low-income elderly groups from purchasing care services [13]; the deep-rooted influence of traditional filial piety makes many elderly people who need formal care reject institutional providers and choose informal care provided by family [14]; deviations in the quality of care services with the impact of marketization lead to a lack of trust to private providers among the elderly [15]; the unpredictability of the elderly’s future morbidity and disease types increases the diversity of care service preference [16].

Actual demand and revenue are closely linked in the market, so uncertain actual demand negatively affects the interests of the investors, which increases the threshold of immediate investment. While many studies have confirmed and emphasized that the initial development of the ECSPs is inseparable from the government’s subsidy incentive and policy guidance, it is obviously not wise for the government to ignore the subsidy costs of the ECSPs [17–19]. Achieving the set goals with the least amount of subsidies should be the concern of the government in policy selection process. In the context of uncertain actual demand, the optimal subsidy scheme should balance the interests of the government and the investors to maximize the policy effects of subsidies, that is, to encourage the investors to invest in the ECSPs immediately while saving unnecessary policy costs. Therefore, it is necessary to compare the policy effects of construction subsidy and operating subsidy to provide constructive opinions for the government to choose the optimal subsidy scheme.

Most of the existing studies discussed the benefit optimization of stakeholders in the ECSPs from the perspective of financing model [20], profit model [21] and management model [22]. In the process of searching for the optimal subsidy scheme, many researchers ignored the influence of actual demand on the stakeholders’ decision-making. Although some scholars used Monte Carlo simulation integrated model [23], discrete event simulation model [24] and robust optimization model [25] to describe, predict and resist the uncertainty of demand respectively, they did not focus on the issue of private investment decision-making. In the field of investment, discounted cash flow model and net present value model were applied in many studies to estimate the value of investment options [26, 27]. However, neither of the two models is applicable to investigate dynamic conditions, and they are limited to situations without uncertainty [28]. Few studies have explored the policy efficiency of subsidy schemes for the ECSPs in stimulating the investors’ immediate investment from the perspective of uncertain actual demand.

Real options model is more suitable for the real market and can deal with the issue of strategy selection under the uncertain situation [29, 30]. It has become a common approach to evaluate available options for the investors and to optimize the time of decision-making in the condition of uncertain demand [31, 32]. With the rise of emerging industries, it has also been employed to optimize the government’s policies, such as the choices of subsidy schemes and the measures of price controls in the new energy industry [33, 34]. Our study employed the real options theory to analyse the difference between construction subsidy and operating subsidy in the realization of policy objectives under the situation of uncertain actual demand, and put forward suggestions on subsidy selection and mechanism optimization. Based on China’s policy experience, the results theoretically clarified the impact of uncertain actual demand on the interests of the government and the investors, and provided a practical reference for the optimal selection of subsidy schemes for the ECSPs in China and other developing countries.

Following the description of subsidy policies, development bottlenecks and investment thresholds of the ECSPs, the real options model was constructed and the mathematical results within the two schemes of construction subsidy and operating subsidy were presented. Then, numerical examples were introduced to identify the optimal subsidy scheme, and the data from the Chinese Longitudinal Healthy Longevity Survey (CLHLS) was embedded to verify the uncertainty of actual demand. Finally, policy implications and limitations of the findings were discussed, and main conclusions of the study were summarized.

### The ECSPs in China

#### Subsidy policies for the ECSPs

**Fig. 1:**
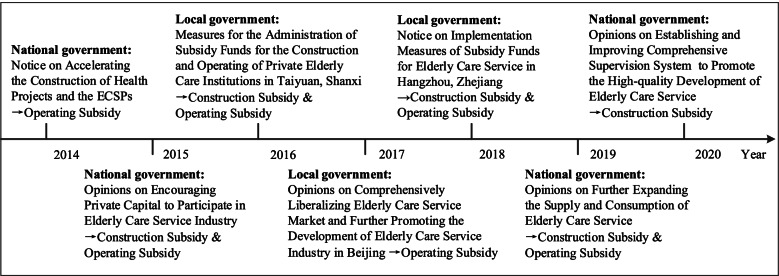
National and local governments’ subsidy policies for the investors in the ECSPs in China [36–39]

As the gap between supply and demand continues to widen, relying solely on the financial strength from the government is insufficient to satisfy the huge demand of the elderly for professional and diversified care services [10]. The fundamental idea of supply-side reform in the healthcare field is to increase the diversity of providers and shift to a market-oriented supply system [35]. Faced with the grim situation of elderly care services insufficiency, the Chinese government adopts the supply-side reform model to encourage private investors to actively participate in the provision of elderly care services. The national and local governments have introduced a series of subsidy policies to motivate the investors to invest in the ECSPs in recent years. As shown in Fig. 1, these subsidy policies mainly include construction subsidy and operating subsidy: the former is a one-time subsidy on a per-bed basis and the latter is a price subsidy on a per-elder basis [11]. The policies aim to achieve that an elderly person with the actual care services demand can get a nursing bed provided by the investors.

The national government mainly plays a guiding or commanding role in the formulation of subsidy policies, and the subsidy funds in the implementation stage come from the local government finance. Therefore, variations in the levels of local economic development lead to variations in the standards and priorities of subsidies [12, 17]. Although there are regional distinctions in subsidies, they share the same mission: to stimulate the investors’ immediate investment while reducing unnecessary subsidy costs. Studies have shown that various subsidy schemes can encourage the private investment and promote the development of healthcare industry, but it remains ambiguous which scheme is more efficient in motivating the investors’ immediate participation [19, 40]. To achieve the goals of subsidy policies, it is necessary to distinguish the policy effects of construction subsidy and operating subsidy, so as to offer implications for the government to choose the optimal subsidy scheme.

#### Development bottlenecks of the ECSPs

**Fig. 2:**
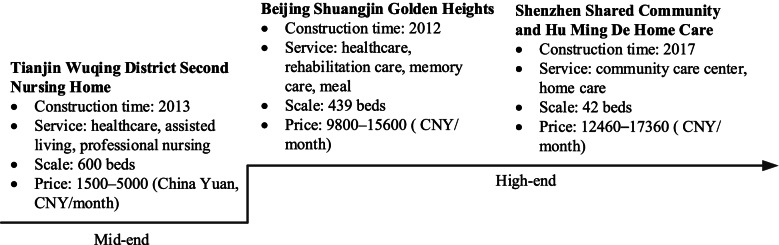
Examples of the ECSPs in China [9]

The theory behind marketization is that competition can force providers to develop products that are cheaper, more flexible and more consumer-oriented. In the United Kingdom, the United States and Germany, most providers of elderly care services are privately-owned [6, 7]. However, the development of China’s ECSPs is still in the stage of “crossing the river by feeling the stones” [6]. The ECSPs have the characteristics of high initial investment and long payback period. In the absence of subsidy incentives, the financial pressure on private investors will extinguish their enthusiasm for investment. And the uncertainty of actual demand for elderly care services directly has a negative impact on the cash flow of investment. A clear market positioning may help the investors set the types of service products for specific customer groups, thereby ensuring the profitability of the projects. In China, commercial institutions are more willing to provide professional elderly care services for mid- to high-end groups in modern cities such as Tianjin, Beijing and Shenzhen, as shown in Fig. 2.

However, the ECSPs have both commercial and public attributes. When formulating subsidy policies, the government should also consider how to encourage the investors to expand the scope of service supply, so as to establish an inclusive elderly care service supply system. Subsidy policies do encourage some investors to invest in the ECSPs, but at present, the scope of the elderly groups served by these projects is limited and the quality of care services provided varies greatly [18]. The inherent profit-seeking motives of private investors make it easy for them to neglect or intentionally reduce the quality of products in the face of commercial interests. Many studies have emphasized the importance of quality supervision of the ECSPs and have explored some practical measures, such as optimizing the *ex ante* control mechanism [41], diversifying the supervision subjects [42] and reducing the information asymmetry between supply and demand [43]. Therefore, appropriate selection and rational design of subsidy schemes are very necessary to realize the industrialization and sustainable development of the ECSPs.

#### Investment thresholds of the ECSPs

**Fig. 3:**
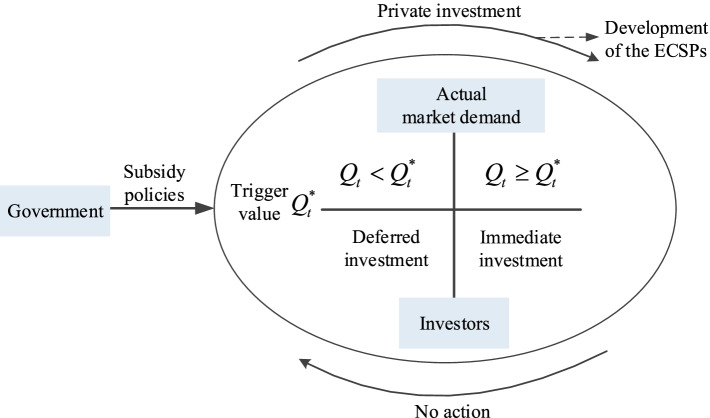
Intervention process of subsidy policies for the ECSPs in China

Although the demand for elderly care services has the huge overall scale and growth potential, the uncertainty of actual market demand makes the economically or financially rational investors postpone investment decisions and take a wait-and-see attitude to ensure that the benefits of investment will not be lost. As shown in Fig. 3, when the actual demand *Q*_*t*_ is less than the trigger value ${Q}_t^{\ast }$, i.e., ${Q}_t<{Q}_t^{\ast }$, the investors will delay investment decisions; only when ${Q}_t\ge {Q}_t^{\ast }$, will investment decisions be exercised immediately. Subsidies are designed to decrease the threshold of investment ${Q}_t^{\ast }$, thereby motivating the investors to participate immediately. From the government’s standpoint, the public benefits derived from the investors’ immediate investment should offset the costs of subsidies. As a result, excessive subsidies should be revised to prevent unnecessary financial burdens, especially for the areas that are relatively underdeveloped. The key point for policymakers is to seek a subsidy scheme in the context of uncertain actual demand, which can stimulate immediate investment and save subsidy costs.

Real options theory is derived from an options valuation technique [44, 45]. It is essentially a stochastic dynamic framework which involves three assumptions: market uncertainty, cost irreversibility and delay ability [46]. This theory recognizes the option value of waiting and emphasizes the flexibility of management [46]. Its model considers investment timing and opportunity costs which are not included in traditional methods, such as discounted cash flow model and net present value model [47]. Many researchers have used it under uncertain conditions to analyse the equilibrium strategies of stakeholders [48, 49], compare the implementation effects of different policies [50, 51] and optimize the decision-making of the investors [52, 53]. In the context of uncertain actual demand, our study also employed the real options theory to explore the difference between construction subsidy and operating subsidy of the ECSPs in achieving the policy goals, and then provided practical implications for the government’s policy choices.

### Model

#### Basic real options model

Considering that the ECSPs can be irreversibly initiated at time *t*, the investment costs *I*_*t*_ can be treated as sunk costs, and the construction of the projects is considered to be completed immediately [46]. Since the benefits of the ECSPs are dependent on the cash flow *W*_*t*_ during the operation time ∆*t*, it is plausible to use the expectation *E*[∙] to express these benefits. The investors in the ECSPs are risk neutral. When they invest in the ECSPs at time *t*, the net expected value of the projects *V*(∙) can be formulated under the risk-neutral probability measure:

1 $$V\left({Q}_t\right)=E\left[{\int}_t^{t+\Delta t}{e}^{-r\left(s-t\right)}{W}_s ds-\mu {I}_t\right]$$

where *r* is the discount rate or the risk-free interest rate, and *μ* is the premium cost coefficient associated with the physical health status of the elderly receiving care services, because the costs of providing services for self-care, semi-disabled and disabled elderly are different [12].

The ECSPs have both commercial and public attributes, so that the price of elderly care services can be regulated and controlled by the government. Therefore, the uncertainty of price is less than that of actual market demand. For simplicity and without loss of generality, the investors are assumed to be price takers. As an elderly person with the actual care services demand should get a nursing bed provided by the investors at the policy level, the actual demand of the elderly is described as the actual demand for nursing beds in this study. If no subsidy is provided, the cash flow *W*_*t*_  is the product of the price of elderly care services *P* and the actual demand *Q*_*t*_ , which can be expressed as:

2 $${W}_t=P\bullet {Q}_t$$

The elderly’s actual demand for institutional care services is uncertain, which is affected by many factors, such as economic conditions, cultural biases and health status. Geometric Brownian motion (GBM) is a common technique used by researchers to model the changing process of demand [54–56]. Our study also employed the GBM to describe the uncertain actual demand *Q*_*t*_ faced by the investors in the ECSPs. The expression can be presented as:

3 $$d{Q}_t=\alpha {Q}_t dt+\sigma {Q}_t dz(t)$$

where *α* and *σ* are risk-neutral drift and volatility of actual demand respectively, *dz*(*t*) is the increment of the Wiener process, $dz(t)={\varepsilon}_t\sqrt{dt}$, and *ε*_*t*_ is a normally distributed random variable with zero mean and unit standard deviation, i.e., *ε*_*t*_~*N*(0, 1). For the convenience of solving the model, our study supposed that the operation time of the ECSPs is the whole lifetime *T* (*T* ≥ ∆*t*) and the construction of the projects can be completed at time *t*_0_. Then, the expected value of *Q*_*t*_ can be obtained as:

4 $$E\left({Q}_t\right)={Q}_{t0}\bullet {e}^{\alpha t}$$

Faced with the uncertainty of actual demand, the economically or financially rational investors can decide whether to invest immediately or to postpone the investment to obtain a more stable cash flow [46]. Namely, they have an investment option and its value *F*(∙) can be given as:

5 $$F\left({Q}_t\right)=\left\{\begin{array}{c}\underset{0\le t\le t+\Delta t}{\mathit{\max}}\left[\mathit{\max}\left(V\left({Q}_t\right),0\right)\right]\kern1em {Q}_t<{Q}_t^{\ast}\kern0.5em \\ {}V\left({Q}_t\right)\kern9.25em {Q}_t\ge {Q}_t^{\ast }\ \end{array}\right.$$

The value of the option to invest in the ECSPs *F*(*Q*_*t*_) will not produce any cash flow until investment decisions are implemented. The investment will occur only when the actual demand reaches or exceeds the trigger value. That is, unless $\kern0.5em {Q}_t\ge {Q}_t^{\ast }$, investment decisions will always be delayed by the investors. Returns of holding investment opportunity in *dt* is the capital expected increment rate *r*. *F*(*Q*_*t*_) should meet the Bellman Equation as below [46]:

6 $$rF\left({Q}_t\right) dt=E\left[ dF\left({Q}_t\right)\right]$$

According to the Ito’s Lemma, *dF*(*Q*_*t*_) is expanded and the differential equation can be derived from Eq. (3) [30]. Then, the following equation is obtained as:

7 $$\frac{1}{2}{\sigma}^2{Q}_t^2\frac{\partial^2F\left({Q}_t\right)}{\partial {Q}_t^2}+\left(r-\delta \right){Q}_t\frac{\partial F\left({Q}_t\right)}{\partial {Q}_t}- rF\left({Q}_t\right)=0$$

where *δ* = *r* − *α* is the rate of return shortfall, which stands for the income gap between holding and exercising the option. *δ* = 0 means that holding the option has no opportunity cost and the investors are unlikely to invest, so our study assumed that *r* > *α*.

The general solution of Eq. (7) takes the form $F\left({Q}_t\right)={A}_1{Q}_t^{\beta_1}+{A}_2{Q}_t^{\beta_2}$, where *β*_1_ and *β*_2_ are positive and negative solutions of the following equation:

8 $$\frac{1}{2}{\sigma}^2\beta \left(\beta -1\right)+\left(r-\delta \right)\beta -r=0$$

which yields:

9 $$\left\{\begin{array}{c}{\beta}_1=\frac{1}{2}-\frac{\alpha }{\sigma^2}+\sqrt{{\left(\frac{\alpha }{\sigma^2}-\frac{1}{2}\right)}^2+\frac{2r}{\sigma^2}}>1\\ {}{\beta}_2=\frac{1}{2}-\frac{\alpha }{\sigma^2}-\sqrt{{\left(\frac{\alpha }{\sigma^2}-\frac{1}{2}\right)}^2+\frac{2r}{\sigma^2}}<0\kern0.5em \end{array}\right.\kern1em$$

To ensure that starting to invest is optimized, the Eq. (7) should also satisfy the following boundary conditions:

10 $$\left\{\begin{array}{c}\underset{Q_t\to 0}{\lim }F\left({Q}_t\right)=0\kern4em \\ {}F\left({Q}_t^{\ast}\right)=V\left({Q}_t^{\ast}\right)\kern3.25em \\ {}{F}^{\prime}\left({Q}_t^{\ast}\right)={V}^{\prime}\left({Q}_t^{\ast}\right)\kern2.5em \end{array}\right.\leftrightarrow \kern0.5em \left\{\begin{array}{c}{A}_2=0\kern9.5em \\ {}{A}_1{Q}_t^{\ast {\beta}_1}=\frac{P{Q}_t^{\ast}\left(1-{e}^{-\delta T}\right)}{\delta }-{\mu I}_t\kern1em \\ {}{\beta}_1{A}_1{Q}_t^{\ast {\beta}_1-1}=\frac{P\left(1-{e}^{-\delta T}\right)}{\delta}\kern1.75em \end{array}\right.$$

Equations $\underset{Q_t\to 0}{\lim }F\left({Q}_t\right)=0$ and $F\left({Q}_t^{\ast}\right)=V\left({Q}_t^{\ast}\right)$ represent the value-matching condition, which means that the option value will be equal to the net expected value when the option is exercised. Equation ${F}^{\prime}\left({Q}_t^{\ast}\right)={V}^{\prime}\left({Q}_t^{\ast}\right)$ represents the smooth-pasting condition, which means that the option value is smooth and continuous. According to these boundary conditions, the minimum critical value ${Q}_t^{\ast }$ (trigger/threshold value) that triggers the investors to invest in the ECSPs can be obtained as:

11 $${Q}_t^{\ast }=\frac{\beta_1}{\beta_1-1}\bullet \frac{\delta \mu {I}_t}{P\left(1-{e}^{-\delta T}\right)}$$

and then the value of the option to invest in the ECSPs *F*(*Q*_*t*_) can be given as:

12 $$F\left({Q}_t\right)=\left\{\begin{array}{c}\frac{\mu {I}_t}{\beta_1-1}{\left(\frac{Q_t}{Q_t^{\ast }}\right)}^{\beta_1}\kern4.00em {Q}_t<{Q}_t^{\ast}\kern1.5em \\ {}\frac{PQ_t\left(1-{e}^{-\delta T}\right)}{\delta }-\mu {I}_t\ \kern1.25em {Q}_t\ge {Q}_t^{\ast}\kern1.25em \end{array}\right.$$

When ${Q}_t<{Q}_t^{\ast }$, the value of the option to invest in the ECSPs *F*(*Q*_*t*_) will be the value to wait; when ${Q}_t\ge {Q}_t^{\ast }$, investment decisions will be executed, and *F*(*Q*_*t*_) will become the net expected value. Therefore, if actual demand is sufficient to induce investment, the value function (payoff) of investment can be described as:

13 $$V\left({Q}_t\right)=E\left[{\int}_0^T{e}^{- rt}{W}_t dt-{\mu I}_t\right]=\frac{PQ_t\left(1-{e}^{-\delta T}\right)}{\delta }-{\mu I}_t$$

#### Real options model embedded subsidies

**Scheme 1** Construction subsidy

Construction subsidy is a one-time subsidy on a per-bed basis. An adequate construction subsidy, fixed in the initial stage of the ECSPs, can encourage the investors to execute their investment decisions. When construction subsidy is provided, the investment costs ${I}_t^{\prime }$ will be composed by the initial input costs *I*_*t*_ and the amount of subsidy actually received, which can be described as:

14 $${I}_t^{\prime }={I}_t-\rho {N}_s{S}_{cs}$$

where *S*_*cs*_ denotes the amount of construction subsidy for each nursing bed, *N*_*s*_ represents the construction or investment scale, i.e., the number of nursing beds supplied by the investors, and *ρ* (0 ≤ *ρ* ≤ 1) is the subsidy adjustment coefficient affected by the government’s supervision on construction quality.

In this scheme, the trigger value ${Q}_{cs}^{\ast }$ is converted to:

15 $${Q}_{cs}^{\ast }=\frac{\beta_1}{\beta_1-1}\bullet \frac{\delta \mu \left({I}_t-\rho {N}_s{S}_{cs}\right)}{P\left(1-{e}^{-\delta T}\right)}$$

Through replacing ${Q}_{cs}^{\ast }$ with *Q*_*t*_ in Eq. (15), *S*_*cs*_ is derived as:

16 $${S}_{cs}=\left\{\begin{array}{c}\frac{I_t}{\rho {N}_s}-\frac{\beta_1-1}{\beta_1}\bullet \frac{PQ_t\left(1-{e}^{-\delta T}\right)}{\rho {N}_s\delta \mu}\kern1.75em {Q}_t<{Q}_t^{\ast}\\ {}0\kern12em {Q}_t\ge {Q}_t^{\ast}\end{array}\right.$$

the value of the option to invest in the ECSPs *F*_*cs*_(*Q*_*t*_) can be given as:

17 $${F}_{cs}\left({Q}_t\right)=\left\{\begin{array}{c}\frac{\mu \left({I}_t-\rho {N}_s{S}_{cs}\right)}{\beta_1-1}{\left(\frac{Q_t}{Q_{cs}^{\ast }}\right)}^{\beta_1}\kern7.00em {Q}_t<{Q}_{cs}^{\ast}\\ {}\frac{PQ_t\left(1-{e}^{-\delta T}\right)}{\delta }-\mu \left({I}_t-\rho {N}_s{S}_{cs}\right)\kern1.75em \ {Q}_t\ge {Q}_{cs}^{\ast}\end{array}\right.$$

and the net expected value of the ECSPs *V*_*cs*_(*Q*_*t*_) can be calculated as:

18 $${V}_{cs}\left({Q}_t\right)=\frac{PQ_t\left(1-{e}^{-\delta T}\right)}{\delta }-\mu \left({I}_t-\rho {N}_s{S}_{cs}\right)$$

**Scheme 2** Operating subsidy

Operating subsidy is a price subsidy on a per-elder basis. When it is provided to the investors, the cash flow of the ECSPs will be increased to ${W}_t^{\prime }$ which is composed by the conventional cash flow *W*_*t*_ and the amount of subsidy actually received. ${W}_t^{\prime }$ can be represented by:

19 $${W}_t^{\prime }=\left(P+\theta {S}_{ps}\right){Q}_t$$

where *S*_*ps*_ denotes the amount of operating subsidy for caring each elderly, *θ* (0 ≤ *θ* ≤ 1) is the subsidy adjustment coefficient affected by the government’s supervision on service quality.

In this scheme, the trigger value ${Q}_{ps}^{\ast }$ is converted to:

20 $${Q}_{ps}^{\ast }=\frac{\beta_1}{\beta_1-1}\bullet \frac{\delta \mu {I}_t}{\left(P+\theta {S}_{ps}\right)\left(1-{e}^{-\delta T}\right)}$$

Through replacing ${Q}_{ps}^{\ast }$ with *Q*_*t*_ in Eq. (20), *S*_*ps*_ is derived as:

21 $${S}_{ps}=\left\{\begin{array}{c}\frac{\beta_1}{\beta_1-1}\bullet \frac{\delta {\mu I}_t}{\kern1.25em {\theta Q}_t\left(1-{e}^{-\delta T}\right)}-\frac{P}{\theta}\kern1.75em {Q}_t<{Q}_t^{\ast}\\ {}0\kern12em {Q}_t\ge {Q}_t^{\ast}\end{array}\right.$$

the value of the option to invest in the ECSPs *F*_*ps*_(*Q*_*t*_) can be given as:

22 $${F}_{ps}\left({Q}_t\right)=\left\{\begin{array}{c}\frac{\mu {I}_t}{\beta_1-1}{\left(\frac{Q_t}{Q_{ps}^{\ast }}\right)}^{\beta_1}\kern7.50em {Q}_t<{Q}_{ps}^{\ast}\\ {}\frac{Q_t\left(P+\theta {S}_{ps}\right)\left(1-{e}^{-\delta T}\right)}{\delta }-{\mu I}_t\kern2.25em {Q}_t\ge {Q}_{ps}^{\ast}\end{array}\right.$$

and the net expected value of the ECSPs *V*_*ps*_(*Q*_*t*_) can be calculated as:

23 $${V}_{ps}\left({Q}_t\right)=\frac{Q_t\left(P+\theta {S}_{ps}\right)\left(1-{e}^{-\delta T}\right)}{\delta }-\mu {I}_t$$

##### Findings

The government’s quality supervision plays an important role in the construction and development of the ECSPs. In Scheme 1 and Scheme 2, the larger values of subsidy adjustment coefficients *ρ* and *θ* are, the more beneficial they will be to decrease the trigger value ${Q}_t^{\ast }$ and subsidy level *S*_*cs*_ or *S*_*ps*_, and to increase the net expected value of the ECSPs *V*(*Q*_*t*_).

The investors’ market positioning for the consumer group affects the option value and investment thresholds. The premium cost coefficient *μ* associated with the physical health status of the elderly receiving care services, the value of the option to invest in the ECSPs *F*(*Q*_*t*_) (${Q}_t<{Q}_t^{\ast }$) and the trigger value ${Q}_t^{\ast }$ show a positive correlation.

Subsidy policies have positive effects on encouraging the investors to implement their investment decisions immediately. Both construction subsidy and operating subsidy contribute to decreasing the trigger value ${Q}_t^{\ast }$. To identify the optimal subsidy from the two schemes, our study introduced numerical examples in the next part.

### Numerical examples

#### Data and assumptions

Since exogenous factors such as market management forms and land use policies have regional disparities, our study didn’t use the cost-benefit data of project cases in a certain area, but chose the simplified and generalized data to simulate a common project case. It makes the model more reproducible and adaptable, and focuses on the uncertainty of actual demand and the optimal selection of subsidy schemes. Table 1 shows the input data used to visualize the model results. The values of these variables and parameters are collected and compiled from previous studies, governmental documents and existing databases.

**Table 1:** Input data for variables and parameters

Variables/Parameters	Description	Units	Values
*I*_*t*_	Investment costs	CNY	60,000,000
*P*	Price of elderly care services	CNY/year	36,000
*T*	Whole lifetime of the ECSPs	year	30
*N*_*s*_	Construction or Investment scale	bed	300
*S*_*cs*_	Construction subsidy	CNY/bed	12,000
*S*_*ps*_	Operating subsidy	CNY/person/year	4800
*ρ*	Construction subsidy adjustment coefficient	_	0.90
*θ*	Operating subsidy adjustment coefficient	_	0.90
*μ*	Premium cost coefficient	_	1.20
*r*	Risk-free interest rate	_	0.10
*α*	Risk-neutral drift rate	_	0.005
*σ*	Risk-neutral volatility rate	_	0.12

The data on costs, prices, lifetime and scale involved in the ECSPs are collected from relevant studies [9, 57, 58], and the amount of subsidies is based on some policies of local governments [36, 38]. Considering that the government can adjust subsidies according to the quality of projects and services, adjustment coefficients of construction subsidy and operating subsidy are respectively assumed: *ρ* = 0.9, *θ* = 0.9. By collecting existing numerical examples, the premium cost coefficient can be calculated as *μ* = 1.2 [43]. Referring to similar studies, the risk-free interest rate is set as *r* = 0.1 [59, 60].

According to the studies on the demand under the GBM characteristics, the values of risk-neutral drift rate *α* and volatility rate *σ* can be estimated using the Maximum Likelihood Estimation [60–62]. The risk-neutral drift rate is calculated by *ω*_*i*_ = (*Q*_*t*_ − *Q*_*t* − ∆*y*_)/*Q*_*t* − ∆*y*_ and $\alpha =\overline{\omega}/\Delta y$, where $\overline{\omega}$ is the mean of  *ω*_*i*_, *Q*_*t*_ represents the variable value at the end of one period, and ∆*y* denotes the time interval in units of years. The risk-neutral volatility rate is calculated by $k=\sqrt{1/\left(n-2\right)\bullet {\sum}_{i=1}^{n-1}{\left({\omega}_i-\overline{\omega}\right)}^2}$ and *σ* = *k*/∆*y*, where *n* is the number of observations.

The CLHLS covers the largest number of the oldest-old in China, which is conducted in half of cities and counties in 23 provinces throughout China. The reliability and validity of the data from the CLHLS have been proven by many studies in the field of health and aging [63–65]. Our study used its eight waves of publicly released datasets comprising the years of 1998, 2000, 2002, 2005, 2008, 2011, 2014, and 2018 to estimate the drift and volatility terms. Since proportional data can reflect the overall change characteristics of the data more comprehensively than numerical data, the proportion of elderly people living in nursing homes in each wave of survey is converted into *Q*_*t*_ as shown in Table 2. Then, *α* = 0.005 and *σ* = 0.12 can be obtained.

**Table 2:** The relevant data from the CLHLS

Year (∆*y* ≈ 3)	Total valid samples	Living in nursing homes	Proportion (% → *Q*_*t*_)	*ω*_*i*_ (*n* = 8)	${\left({\omega}_i-\overline{\omega}\right)}^2$
1998	9093	457	5.03	_	_
2000	11,199	784	7.00	0.39	0.14
2002	16,064	740	4.61	−0.34	0.13
2005	15,638	422	2.70	−0.41	0.19
2008	16,954	308	1.82	−0.33	0.12
2011	9638	207	2.15	0.18	0.03
2014	7020	198	2.82	0.31	0.09
2018	15,549	574	3.69	0.31	0.09

#### Numerical results

**Fig. 4:**
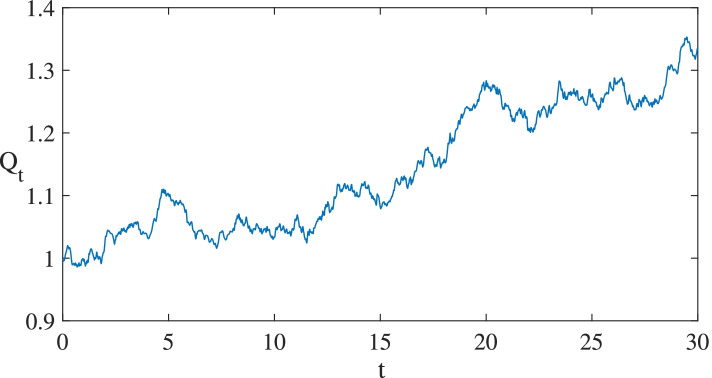
Sample path of actual demand

Matrix Laboratory software is used to solve the model and present the outcomes. Figure 4 simulates the uncertain actual demand with time *t* on the x-axis and actual demand *Q*_*t*_ on the y-axis. The path of *Q*_*t*_ is plotted by embedding the value group of (*α* = 0.005, *σ* = 0.12). It clearly shows that the actual demand is rising in volatility. Since the actual demand directly affects the cash flow obtained by the investors, the ECSPs will be an attractive investment option when the actual demand reaches or exceeds a certain level.

**Fig. 5:**
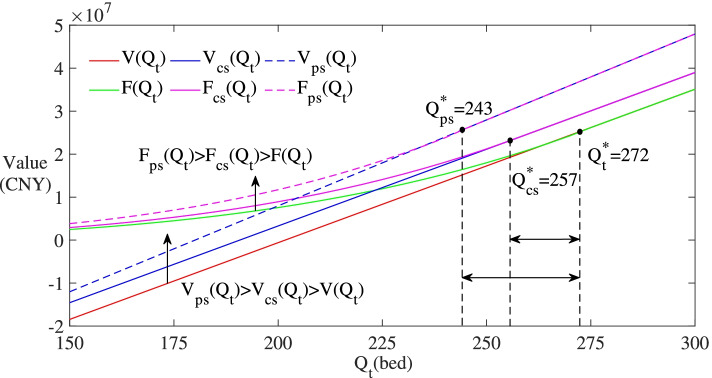
Results in Scheme 1 and Scheme 2

Figure 5 sets the values of *F*(∙) and *V*(∙) on the y-axis and selects *Q*_*t*_ ∈ (150,300) on the x-axis. The trigger value of investment is determined by the tangency point of *F*(∙) and *V*(∙). When no subsidy is provided, its value is ${Q}_t^{\ast }=272$; when subsidies are provided in Scheme 1 and Scheme 2, the tangency point moves to the left and the value decreases to ${Q}_{cs}^{\ast }=257$ and ${Q}_{ps}^{\ast }=243$ respectively. Moreover, the curves of *F*(∙) and *V*(∙) move up in both schemes. Then, ${Q}_t^{\ast }>{Q}_{cs}^{\ast }>{Q}_{ps}^{\ast }$, *F*_*ps*_(*Q*_*t*_) > *F*_*cs*_(*Q*_*t*_) > *F*(*Q*_*t*_) and *V*_*ps*_(*Q*_*t*_) > *V*_*cs*_(*Q*_*t*_) > *V*(*Q*_*t*_) are obtained.

The results show that subsidies can decrease the trigger value of investment, increase the option value and the net expected value of the ECSPs. In other words, subsidies can act as incentives for the investors to invest immediately. However, different forms of subsidies can lead to different policy effects. The choice of subsidy schemes is important for policymakers to achieve the established goals. According to the results shown in Fig. 5, operating subsidy is more effective than construction subsidy in this regard.

**Fig. 6:**
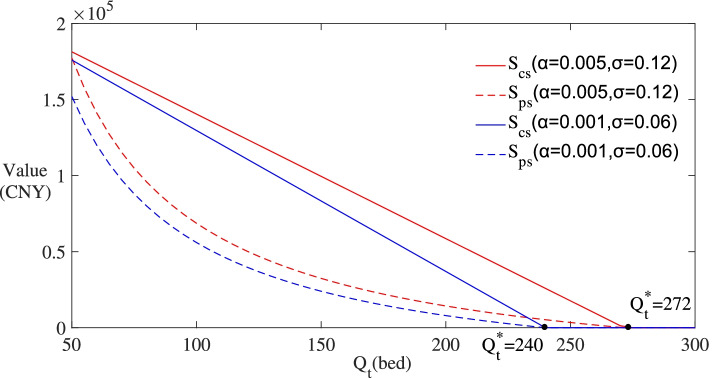
Changes of subsidies with the uncertainty of actual demand

Figure 6 depicts the subsidy value on the y-axis and the actual demand *Q*_*t*_ ∈ (50,300) on the x-axis. To highlight the fluctuation of actual demand, (*α* = 0.001, *σ* = 0.06) is added as a reference group. As the values of *α* and *σ* decrease, the curves of *S*_*cs*_ and *S*_*ps*_ move down accordingly, and the trigger value of investment decreases from ${Q}_t^{\ast }=272$ to ${Q}_t^{\ast }=240$. Therefore, from the perspective of saving policy costs and stimulating immediate investment, the uncertainty of actual demand should be timely concerned by the government when formulating subsidy policies.

The curves of *S*_*cs*_ and *S*_*ps*_ show a downward trend with the actual demand increasing. When the actual demand reaches the investment threshold ${Q}_t^{\ast }$, the values of *S*_*cs*_ and *S*_*ps*_ drop to zero. In this process, operating subsidy is always lower than construction subsidy, which explains that operating subsidy is more conducive to saving policy costs. Therefore, in the context of uncertain actual demand, the choice of subsidy schemes does have an impact on policy costs. The results also conform that when the actual demand reaches the trigger value, subsidies will no longer be needed.

**Fig. 7:**
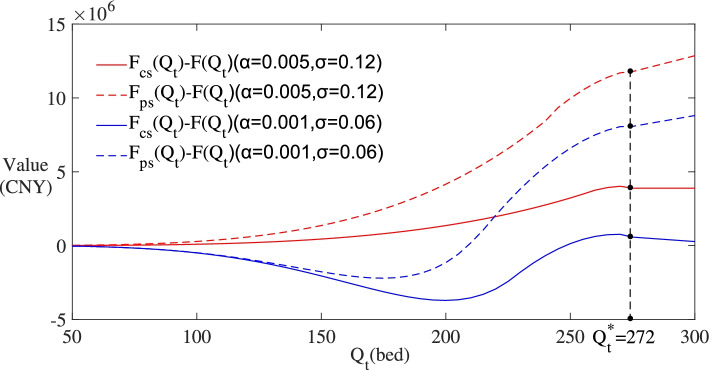
Changes of subsidies’ effects on *F*(∙) with the uncertainty of actual demand

**Fig. 8:**
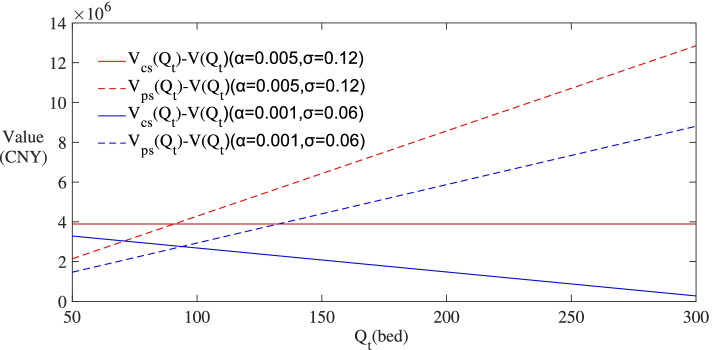
Changes of subsidies’ effects on *V*(∙) with the uncertainty of actual demand

To display the spillover effects of subsidies on *F*(∙) and *V*(∙), Figs. 7 and 8 set the values of *F*_*cs*_(*Q*_*t*_) − *F*(*Q*_*t*_), *F*_*ps*_(*Q*_*t*_) − *F*(*Q*_*t*_) , *V*_*cs*_(*Q*_*t*_) − *V*(*Q*_*t*_) and *V*_*ps*_(*Q*_*t*_) − *V*(*Q*_*t*_) on the y-axis. These values can be interpreted as the spillover benefits from subsidies for the investors. According to the results in the two figures, the spillover benefits from operating subsidy are always higher than those from construction subsidy with actual demand fluctuating. And as shown in Fig. 8, the curve of *V*_*ps*_(*Q*_*t*_) − *V*(*Q*_*t*_) shows an increasing trend. The results imply that compared with construction subsidy, operating subsidy is more adaptable to the uncertainty of actual demand and is more beneficial to the investors in the long run.

## Discussion

Formulating subsidy policies to promote the development of the ECSPs is an important means for the government to leverage market forces to provide care services for the elderly under the supply-side reform model. Our study applied the real option theory to establish a mathematical model for comparing the effects of subsidy policies, and introduced numerical examples to identify the optimal subsidy scheme and to verify the uncertainty of actual demand. The results showed that operating subsidy is more effective than construction subsidy in stimulating the investors’ immediate investment, saving policy costs and increasing spillover value of subsidy policy. The study not only presented a theoretical framework for analysing the dynamic changes of the investors’ investment strategies for the ECSPs in the face of uncertain actual demand, but also provided a practical and directional reference for the government’s optimal subsidy selection.

For the investors, operating subsidy is more attractive for them to invest in the ECSPs immediately, as it can significantly increase the option value and the net expected value of investment in the context of uncertain actual demand. Moreover, operating subsidy is directly related to the cash flow of the ECSPs, which can effectively alleviate the risk of income caused by the uncertainty of actual demand, thereby enhancing the investors’ investment confidence and driving them to execute investment decisions immediately. According to our research results, the actual demand curve shows an upward trend in fluctuation. Therefore, with the increase of actual demand in the future, the investors will get a higher spillover net expected value from operating subsidy than from construction subsidy, which is beneficial to increase the long-term interests of investing in the ECSPs.

For the government, operating subsidy has greater advantages than construction subsidy in terms of reducing investment thresholds and saving policy costs. Operating subsidy can lower investment thresholds even more, thus triggering immediate investment more quickly. While it is true that throwing substantial financial subsidies into the ECSPs can stimulate a lot of immediate investment, avoiding unnecessary subsidy costs is also a manifestation of the government’s responsibility to the public interests. Operating subsidy appears to be more conducive to achieving the policy goals at the lower policy costs. Moreover, by adding a reference group to the parameter values of uncertainty, the study highlighted the policy adaptability of operating subsidy to the uncertainty of actual demand. Operating subsidy based on actual demand can effectively reduce the number of vacant beds in the ECSPs and encourage the investors to expand the scope of service supply, thereby promoting the ECSPs’ development towards a more inclusive direction. The results emphasized the importance of policy choices.

Additionally, the results showed that the government should pay attention to the quality supervision of the ECSPs, the market positioning of the consumer group and the long-term growth of actual market demand. Measures that can improve the quality supervision system should be taken, especially something regarding medical and health care, such as technical audit of professional nursing equipment and qualification assessment of practitioners [18]. Investment costs vary with the self-care ability of the elderly receiving care services. It is worth popularizing that local governments such as Beijing and Hangzhou have adopted differentiated operating subsidy scheme according to the health statue of the elderly served by the projects [36, 37]. Some policies that can increase the affordability of the elderly should be emphasized, such as improving the level and coverage of old-age insurance, providing payment vouchers for the oldest-old, and establishing a long-term care insurance system [12]. The implementation of these policies can be conducive to creating a sustainable growth market demand environment for the ECSPs.

China’s policy experience provides a paradigm for many developing countries that are devoting to introducing market forces through the ECSPs to provide elderly care services. Our study verified the uncertainty of actual demand and clarified its impact on the interests of the government and the investors in the initial stage of the ECSPs. The results emphasized the importance of subsidy intervention in stimulating immediate investment and the advantages of operating subsidy in the context of uncertain actual demand. However, there are also some limitations that deserve to be improved in the future. Firstly, this study verified the uncertainty of actual demand, but it didn’t analyse the main driving forces behind it in detail to further explore the space of policy optimization. Secondly, the aim of this study is to provide more reproducible and adaptable policy suggestions for the ECSPs, so the regional variables and the data of typical cases are not introduced into the real options model and numerical examples, which can be further explored after more complete and appropriate data are collected.

## Conclusions

Rapidly aging of the population spawns a huge demand for elderly care services. Under the supply-side reform model, motivating private investors to invest in the ECSPs immediately to satisfy the vast demand volumes with fewer subsidy costs is the government’s policy goals. The study showed that operating subsidy is more effective in achieving these policy objectives than construction subsidy in the context of uncertain actual demand. Moreover, a sound quality supervision system, a differentiated operating subsidy scheme and a sustainable growth market demand environment are conducive to increasing the interests of the government and the investors in the long term. The results not only provided directional guidance and opinions for the sustainable development of the ECSPs in China, but also presented empirical paradigms for policy designers in other developing countries that expect to use market forces to deal with the demand of elderly care services.

## Data Availability

The CLHLS data is publicly available. Information about the data can be found at https://opendata.pku.edu.cn/dataset.xhtml?persistentId= 10.18170/DVN/WBO7LK. Researchers can obtain it after submitting a data use agreement to the CLHLS team. The full datasets used in this study are also available from the corresponding author upon reasonable request.

## Abbreviations

ECSPs: Elderly Care Service Projects
CNY: China Yuan
GBM: Geometric Brownian Motion
CLHLS: Chinese Longitudinal Healthy Longevity Survey
